# Prediction of white matter hyperintensities evolution one-year post-stroke from a single-point brain MRI and stroke lesions information

**DOI:** 10.1038/s41598-024-83128-6

**Published:** 2025-01-07

**Authors:** Muhammad Febrian Rachmadi, Maria del C. Valdés-Hernández, Stephen Makin, Joanna Wardlaw, Henrik Skibbe

**Affiliations:** 1 RIKEN Center for Brain Science, Brain Image Analysis Unit, Wako-shi, 351-0106 Japan; 2 Faculty of Computer Science, Universitas Indonesia, Depok, 16424 Indonesia; 3 Centre for Clinical Brain Sciences, University of Edinburgh, Edinburgh, EH16 4SB UK; 4 Centre for Rural Health, University of Aberdeen, Inverness, IV2 3JH UK

**Keywords:** Predictive markers, Stroke

## Abstract

Predicting the evolution of white matter hyperintensities (WMH), a common feature in brain magnetic resonance imaging (MRI) scans of older adults (i.e., whether WMH will grow, remain stable, or shrink with time) is important for personalised therapeutic interventions. However, this task is difficult mainly due to the myriad of vascular risk factors and comorbidities that influence it, and the low specificity and sensitivity of the image intensities and textures alone for predicting WMH evolution. Given the predominantly vascular nature of WMH, in this study, we evaluate the impact of incorporating stroke lesion information to a probabilistic deep learning model to predict the evolution of WMH 1-year after the baseline image acquisition, taken soon after a mild stroke event, using T2-FLAIR brain MRI. The Probabilistic U-Net was chosen for this study due to its capability of simulating and quantifying the uncertainties involved in the prediction of WMH evolution. We propose to use an additional loss called volume loss to train our model, and incorporate stroke lesions information, an influential factor in WMH evolution. Our experiments showed that jointly segmenting the disease evolution map (DEM) of WMH and stroke lesions, improved the accuracy of the DEM representing WMH evolution. The combination of introducing the volume loss and joint segmentation of DEM of WMH and stroke lesions outperformed other model configurations with mean volumetric absolute error of 0.0092 ml (down from 1.7739 ml) and 0.47% improvement on average Dice similarity coefficient in shrinking, growing and stable WMH.

## Introduction

### White matter hyperintensities and their progression

White matter hyperintensities (WMH) are one of the main neuroradiological features of cerebral small vessel disease (SVD) and have been commonly associated with stroke, aging, and dementia progression^1–3^. They are often observed in T2-weighted and T2-fluid attenuated inversion recovery (T2-FLAIR) brain magnetic resonance images (MRI), appearing as bright regions. Small subcortical infarcts may be indistinguishable from WMH on structural MRI in absence of intravenous contrast due to sharing similar image intensity characteristics^4^, and if mistaken for WMH could negatively impact the design of clinical research trials^5^.

Clinical studies have indicated that some patients exhibit WMH progression over time (i.e., increasing in volume)^6–8^ while some show WMH regression over time (i.e., shrinking in volume)^9,10^, although these are fewer in proportion compared to those reporting an increase in volume^10^. Another study indicated that WMH dynamically change over time with clusters of WMH individually shrinking, staying unchanged (i.e., stable), or growing, these being observed at the same time point within the same individual^11^. These variations have been associated with patients’ comorbidities and clinical outcome^3,12^. A meta-analysis on rate and risk factors for WMH volume growth specifically, concluded that these vary with the characteristics of the sample, although hypertension, age, baseline WMH volume and smoking seemed to be the main contributors^13^. And a growing number of clinical studies have indicated that, in addition to age^8^, previous strokes^14^ and genetics^15–18^ also influence the rate and direction of WMH evolution. But, as one clinical study and another meta-analysis acknowledged, current knowledge about factors influencing WMH evolution is still incomplete and poorly understood^3,10^.

Interestingly, despite increasing evidence on WMH burden at baseline being the determinant factor on the rate and magnitude of WMH progression (and regression)^13^, increase in WMH volume has been found to be a better predictor of persistent cognitive impairment (i.e., a potential precursor to Alzheimer or vascular dementia) than baseline WMH burden^19^. However, evidence that overall reduction of WMH volume over time can prevent functional decline is scarce^2^. In terms of spatial WMH evolution, a study on patients that had a mild stroke of type lacunar found that post-stroke cognition at 1 and 3 years was affected by the location of WMH^20^. But despite evidence on the importance and benefit of studying WMH spatial distribution^21^, there are limited approaches to predict spatial WMH evolution.

Predicting the evolution of WMH is crucial for understanding the dynamics of small vessel disease and ultimately provide better care and prognosis for individual patients. It has been suggested that as WMH shrinkage may partially be due to interstitial fluid alterations, analysis of WMH evolution constitute a potential intervention target^3^. A review on associations and implications of WMH growth and shrinkage^13^ mentions that several of the studies reviewed assessed potentially treatable risk factors influencing WMH progression. It mentions that hypertension was reported to be significantly associated with WMH growth in 18 of the 52 studies reviewed, as well as current smoking status, and that modification of these risk factors could improve patient outcome. The same publication notes that early interventions may be more successful than when there may be a level of cognitive impairment which prevents any improvement in WMH evolution from translating into functional benefit^13^. Another study reports that adding WMH volume to statistical models predicting incident or recurrence of stroke or cognitive impairment in hypertensive patients improved the prognostic ability of such models to consistently give an excellent prediction, above predictions that used vascular risk and demographic factors^22^. However, prediction of WMH evolution remains a difficult task because of the different rate and direction of the evolution of individual WMH clusters and their interplay with other imaging features of vascular disease and brain parenchymal changes^14^. Specifically, 1 year after stroke, reported WMH changes are mild^13^, thus posing an additional challenge for their accurate identification.

### Precedent work in estimating WMH evolution

**Fig. 1:**
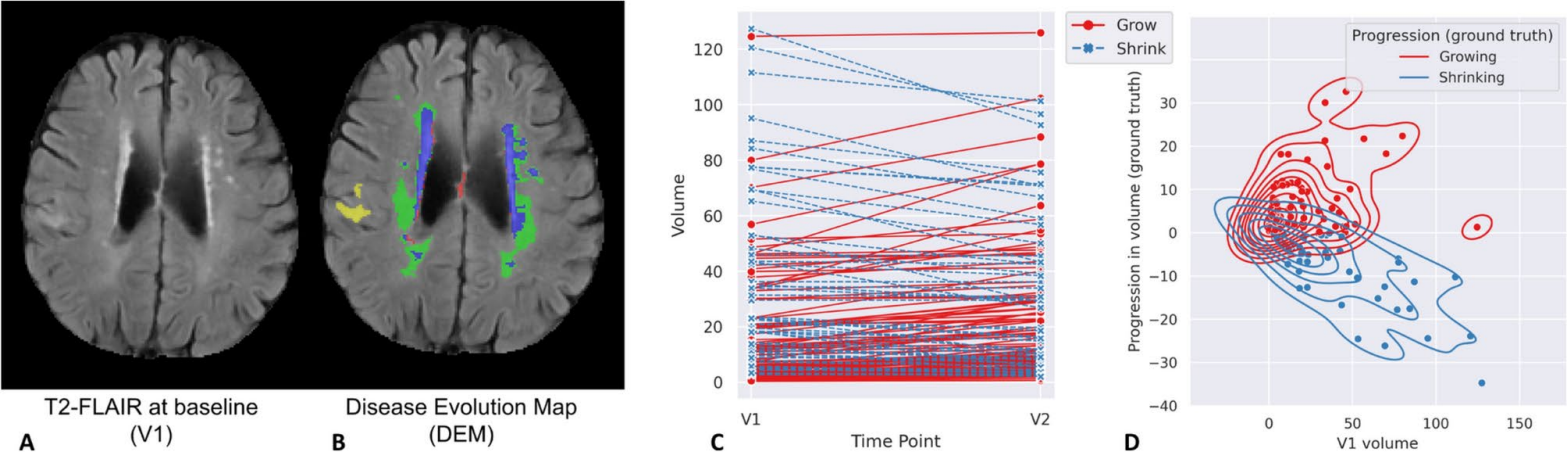
(**A**): Brain-extracted FLAIR axial slice of the baseline scan or V1. (**B**): Visualisation of disease evolution map (DEM) of white matter hyperintesities (WMH). Red represents shrinking WMH, green represents growing WMH, blue represents stable WMH, and yellow represents stroke lesions. (**C**): Volumetric progression of WMH (in ml) from V1 to V2 (1 year apart) for all subjects from our dataset. (**D**): shows the distribution of volumetric progression of WMH (in ml) based on WMH volume at V1 for all subjects.

Despite the high accuracy displayed by several fully-automatic deep learning schemes segmenting WMH^23^, most of the algorithms applied in longitudinal studies on WMH evolution have been so far semi-automatic^13^. Various deep learning models have been proposed to predict the spatial evolution of WMH^24–26^. These studies, have represented WMH spatial evolution by a map called *disease evolution map* (DEM) which indicates the WMH voxels that shrink, grow, or remain stable at a further time point. DEM can be generated by subtracting images of manually labeled WMH from different time points. Previous studies generated the DEM by subtracting a baseline image of semi- or fully-automatically labeled WMH of a patient (Visit 1, V1) from a follow-up image of semi- or fully-automatically labeled WMH from the same patient one year after (Visit 2, V2)^25,26^. An example of DEM is visualised in Fig. 1B.

A recently proposed model for predicting the DEM of WMH based on a Probabilistic U-Net^27^, generates multiple DEM predictions for a single brain MRI data^26^. This model was proposed to solve the challenge of representing spatial uncertainty^25^, given difficulties in distinguishing intensities and textures of shrinking and growing WMH in T2-FLAIR brain MRI. Models using Probabilistic U-Net performed significantly better than the classical U-Net models in predicting the evolution of WMH using DEM^26^.

All these previous approaches have focused, almost exclusively, on the image modality as input and the appearance of WMH themselves, ignoring other clinically relevant factors. A subsequent study incorporated volume of stroke lesions as auxiliary input to the prediction model, but it did not improve the prediction results^25^. Another study^28^ used radiomic signatures of the normal-appearing tissue as auxiliary variables to vascular risk factors in a logistic regression model to predict general “progression” vs. “no progression” of WMH, and reported that radiomics improved the accuracy of the model by approximately 10%, but did not analyse the spatial change of WMH. Thus, incorporating clinically associated factors into the predictive model remains a challenge for estimating the spatial evolution of WMH.

### Related approaches

Studies that develop predictive models for disease progression from medical image modalities using machine/deep learning can be categorised, generally, into the three different approaches listed below.

1. Approaches predicting the outcomes of a disease. These approaches are commonly used for diseases with high rates of mortality and disability. Some examples are those predicting the outcomes of COVID-19^29^, multiple sclerosis^30^, and traumatic brain injury^31,32^.
2. Approaches predicting the progression of a disease with regards to the pathological timeline and/or commonly associated disease markers. These approaches are commonly used for diseases with multiple stages of development and which take time to progress, such as dementia and Alzheimer’s Disease (AD), with mild cognitive impairment (MCI) being their transitional stage^33^. Some examples are predicting conversion of MCI patients to AD^34^, conversion of healthy individuals to MCI and AD^35^, and predicting the progression of multimodal AD markers (e.g., ventricular volume, cognitive scores, etc.)^36^.
3. Approaches predicting dynamic changes (evolution) of specific disease features. These approaches model and predict spatial changes of specific disease features such as evolution of WMH, enlargement of ventricles, and brain atrophy. Other examples are predicting lung nodule progression of pulmonary tumour^37^, predicting dynamic change of brain structures from healthy individuals to MCI and AD patients^38^, and studies for predicting the evolution of WMH in brain images of stroke patients^24–26^

This study belongs to the third category, in which a predictive model is used to spatially estimate the dynamic changes of WMH on an MRI scan at a certain time point. This third category is the most challenging because of the complexity and resolution of the data/image being predicted, especially when the time-point estimated is close to the baseline scan. While approaches in the first and second categories predict classes which are the disease outcomes (e.g., survive, death), classes of disease stages (e.g., MCI, AD), or associated disease markers (e.g., age, cognitive scores) from medical imaging data, approaches in the third category predict the evolution of disease’s imaging features (e.g., lesions and their volumes) spatially, i.e., throughout the entire image space.

### Our contributions

The main contributions of this study are twofold, and show that they considerably improve the prediction of WMH volume and spatial change 1 year after a mild-to-moderate stroke event:

1. incorporating stroke lesions’ information to the prediction model and
2. adding a volume loss to the cost function (formulated as the mean squared error between the predicted and the reference future WMH volumes) to improve prediction of WMH evolution voxel-wise.

As part of a comprehensive set of evaluations, We also evaluate the output from our schemes against the clinical visual scores for WMH evolution^39^, and analyse the degree of uncertainty in our predictions.

### Proposed deep learning model

Uncertainties are unavoidable when predicting the progression of WMH. Previous studies showed that incorporating uncertainties into a deep learning model, either by incorporating Gaussian noise as auxiliary input^25^ or using a conditional variational autoencoder in the shape of a Probabilistic U-Net with adversarial training^26^, improved prediction results, thus justifying the use of a Probabilistic U-Net with adversarial training in the present study.

### Probabilistic U-Net with adversarial training

**Fig. 2:**
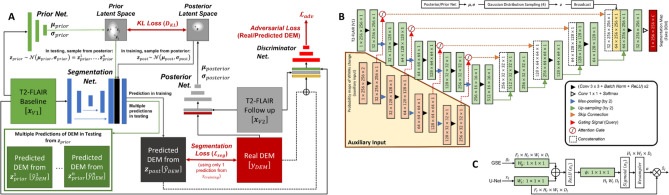
(**A**) Schematic representation of the Probabilistic U-Net^27^ with adversarial training^41^ used in this study, firstly introduced in a previous work^26^. (**B**) Segmentation network of Probabilistic U-Net used in this study, which is based on the original U-Net extended into Attention U-Net only when probability maps of WMH change are used as auxiliary input. The output channel of *C* is either 5 or 4 depending on whether stroke lesions are jointly segmented or not, respectively. (**C**) Schematic of additive attention gate (AG) used in this study, firstly introduced in^42^. Input features (*x*_*l*_) are from the U-Net’s skip connection while gating signals (*g*_*l*_) are from the gating signal encoder (GSE). Attention coefficients (*α*) are learned in the training process and used to scale input features *x*_*l*_ to highlight important areas.

The uncertainty associated with the randomness in the dynamism of the WMH clusters is commonly known as *aleatoric uncertainty*^40^. It constitutes the biggest challenge in predicting WMH evolution, due to differences between experts in WMH delineation (i.e., ground truth reliability issues), and difficulty in differentiating textures and intensities of shrinking and growing WMH in the T2-FLAIR MRI sequence^25^. This uncertainty cannot be reduced by simply adding more training data^40^. The use of a Bayesian deep learning model named Probabilistic U-Net^27^ was previously proposed to overcome this challenge, and generated better prediction results than non-probabilistic models^26^. In this study, we modify the previously proposed approach, as Fig. 2A schematically illustrates.

The probabilistic U-Net with adversarial training consists of a U-Net configuration^43^, two variational encoders called Prior Net and Posterior Net, and a discriminator network for adversarial training. This study used a U-Net as a base segmentation network, and a Probabilistic U-Net for predicting the DEM, as preliminary experiments showed that among U-Net configurations it performed best for generating the DEM^26^. Meanwhile, Prior Net and Posterior Net were used for variational inference so that uncertainty in predicting future WMH evolution is modeled probabilistically. Prior Net estimates a low-dimensional Gaussian distribution called prior latent space by producing its mean(s) and variance(s) from T2-FLAIR MRI at baseline (i.e., V1, denoted *x*_*V*1_). Whereas, Posterior Net estimates another low-dimensional Gaussian distribution called posterior latent space by producing its mean(s) and variance(s) from the follow-up T2-FLAIR MRI (i.e., V2, denoted *x*_*V*2_) and ground truth DEM (*y*_*DEM*_). In reality, the posterior latent space is unknown because the follow-up T2-FLAIR MRI and the ground truth DEM are not present. Because of that, *Kullback–Leibler* divergence is used during training to estimate a posterior latent space from the prior latent space, obtained from the baseline T2-FLAIR MRI. In training, a sample *z*_*post*_ is taken from the posterior latent space (*z*_*post*_ ∼ *N µ*_*post*_*,* *σ*_*post*_) and then broadcasted and concatenated to the segmentation network. Multiple predictions of DEM (${^\wedge} {y^1}_{DEM}$ *, *$\hat{y}_{DEM}^{2} ,\; \cdots$ ${y^\wedge {n}}_{DEM}$ ) can be generated by using multiple samples ($Z_{prior}^{1} ,\;Z_{prior}^{2} ,\; \cdots ,z_{prior}^{n}$*, *) from the prior latent space (*z*_*prior*_ ∼ *N µ*_*prior*_*, σ*_*prior*_). In this study, 30 different DEM predictions were generated from 30 samples of *z*_*prior*_ from Prior Net for each input data/patient in the inference, and then averaged to get the final DEM prediction. Lastly, a discriminator network is used for adversarial training to enforce anatomically realistic DEM from the T2-FLAIR MRI at V1 and V2, similar to previous work^25^. For all network architectures (i.e., the U-Net, Prior Net, Posterior Net, and discriminator) we implemented the configurations that performed best in preliminary experiments^26^. The U-Net, Prior Net, Posterior Net, and discriminator have approximately 31.5M, 18.9M, 18.9M, and 13.3M trainable parameters respectively.

### Incorporation of stroke lesions information

Clinical studies have indicated strong correlations between stroke occurrence and progression of WMH over time^14^. In a previous study^25^, stroke lesion volume was used as an auxiliary input to a framework designed to estimate WMH evolution. However, it was outperformed by using Gaussian noises as auxiliary input representing uncertainty. Thus, in this study, we explore how information on stroke lesions can be incorporated into the Probabilistic U-Net to better predict the future volume of WMH and their evolution. We propose two different approaches: (1) jointly segmenting the WMH DEM and stroke lesions and (2) incorporating probabilistic maps of WMH change in relation to stroke lesions’ locations. The second proposed approach is more complex than the first proposed approach because it needs multiple preprocessing steps.

#### Joint segmentation of DEM and stroke lesions

Due to the similar tissue signal intensity of WMH and ischaemic stroke lesions in T2-FLAIR brain MRI, we hypothesised that performing a joint segmentation of the WMH DEM and stroke lesions will improve the accuracy in the prediction of the WMH DEM because the deep learning model will automatically learn the spatial correlation between both features. In this proposed approach, stroke lesions do not need to be excluded in the preprocessing steps like in preceding works^25,26^. This approach can be implemented by adding an output channel to the segmentation layer of the segmentation network, thus increasing the number of output channels from four channels (i.e., channels for background, shrinking WMH, growing WMH, and stable WMH), to five channels. Note in Fig. 1B that the label ‘stroke lesions’ has been added to the DEM of WMH. In this proposed approach, the generator (i.e., the U-Net) has 12,032 additional trainable parameters compared to the original U-Net.

#### Probabilistic maps of WMH change in relation to stroke lesions’ locations

Results from a clinical study indicate that there are strong correlations between stroke lesions’ location at baseline (V1) and WMH evolution after 1 year (V2)^20^ for patients with a stroke of type lacunar. Specifically, if stroke lesions are subcortical and located in either the *centrum semiovale* or the *lentiform nucleus* at V1, then there are significant changes to the WMH at V2 (both in volume and location) specific to the location of the stroke lesions at V1. This clinical study made available probability maps of WMH change indicating brain locations where changes of WMH are significant at V2 depending on the infarcted region after accounting for vascular risk factors (VRF)^44^.

In this approach, probabilistic maps indicating specific areas of the brain where a previous study showed a statistically significantly faster (or larger) growth of WMH after a lacunar stroke, depending on where the stroke lesion is located (i.e, centrum semiovale or lentiform nucleus), are used as an auxiliary input to an attention U-Net^42^ within the Probabilistic U-Net’s segmentation network. This is illustrated in Fig. 2B, where these probabilistic maps are feed-forwarded through the auxiliary input highlighted in the yellow box. The information of the probability maps is encoded through the gating signal encoder (GSE), illustrated as the yellow box in Fig. 2B, with outputs used as the gating signals to the U-Net’s feature maps in multiple resolutions (see Fig. 2C). The general idea and rationale of this approach is to focus the attention of the segmentation network on the areas that have a high probability of WMH change according to the locations of the stroke lesions. This approach differs from the joint segmentation approach in the additional input provided to the deep learning model. For this approach, we performed brain parcellation and registration of the probability maps (in standard image space) to each patient’s space to identify the locations of stroke lesions for each specific patient. In this setting, the generator has 32.2M trainable parameters (i.e., 725,140 additional trainable parameters compared to the original U-Net).

Similar to the original Attention U-Net^42^, this study uses an additive attention gate (AG), but obtains the gating signals from the GSE instead of from the outputs of the next (coarser) convolutional block. The schematic of the additive AG can be seen in Fig. 2C. Input features (*x*_*l*_) are from the U-Net’s skip connections, gating signals (*g*_*l*_) are from the gating signal encoder (GSE), *α* are the attention coefficients learned in the training process used to scale input features *x*_*l*_ to highlight important areas, ^L^ is an element-wise addition, ^N^ is an element-wise multiplication, and *W*_*g*_, *W*_*x*_, and *ψ* are 1 × 1 × 1 convolution operations.

### Configurations of the proposed approach

In this study, we evaluate four configurations of the Probabilistic U-Net (PUNet) segmentation network. We compared three methods of incorporating probabilistic maps of WMH and/or stroke lesions with the vanilla U-Net as described above. These are: (1) joint segmentation of the DEM of WMH and stroke lesions (i.e., denoted as PUNet-wSL), (2) use of the probabilistic maps of WMH change in relation to stroke lesions’ locations (i.e., denoted as Att-PUNet), and (3) the combination of both methods (i.e., denoted as Att-PUNet-wSL). All of them took 5-8 minutes to train per epoch.

1. UNet: The original U-Net^43^ is used for segmenting the DEM of WMH.
2. UNet-wSL: The original U-Net^43^ is used for joint segmentation of the DEM of WMH and stroke lesions.
3. PUNet: Probabilistic U-Net^27^ with adversarial training^26^ where the original U-Net^43^ is used for segmenting the DEM of WMH.
4. PUNet-wSL: Joint segmentation of the DEM of WMH and stroke lesions is performed by a Probabilistic U-Net^27^ with adversarial training^26^ where the original U-Net^43^ is used for the segmentation network.
5. Att-PUNet: The Probabilistic U-Net’s segmentation network uses the attention U-Net with probabilistic maps of WMH change instead of the original U-Net, for segmenting the DEM of WMH.
6. Att-PUNet-wSL: The Probabilistic U-Net’s segmentation network uses the attention U-Net with probabilistic maps of WMH change instead of the original U-Net, for joint segmentation of the DEM of WMH and stroke lesions.

## Experimental setting

This section describes the dataset, training scheme, cost function, and evaluation metrics this study uses. This study, and the study that provided the data were conducted in accordance with the Declaration of Helsinki.

### Dataset

For comparability of our results with those previously published, we use the same dataset as^25^, which comprises MRI data from *n* = 152 patients that had a mild-to-moderate stroke and gave informed consent to participate in a study of stroke mechanisms^3^. The study protocols were approved by the Lothian Ethics of Medical Research Committee (REC 09/81101/54) and NHS Lothian R+D Office (2009/W/NEU/14), on the 29th of October 2009. All patients were imaged with the same acquisition protocol at two time points (i.e., baseline scan (V1), and a year after the baseline scan (V2)). In total, 304 MRI from 152 stroke patients (i.e., 152 V1 MRI and 152 V2 MRI) were used. Overall increase in WMH volume was identified in 98 of the 152 patients and reduction of WMH total volume in 54 patients. The magnitudes of WMH change (in *ml*) and their distribution for all patients can be seen in Fig. 1C and D.

All T2-FLAIR brain MRI were acquired with a GE 1.5T scanner, and a semi-automatic multi-spectral method was used to produce several brain masks including intracranial volume, cerebrospinal fluid, stroke lesions, and WMH, all which were visually checked and manually edited by an expert^45^. For the prediction of WMH evolution from V1 to V2, T2-FLAIR brain MRI at follow-up (V2) and T2-FLAIR brain MRI at baseline (V1) were linearly and rigidly aligned to a common space using FSL-FLIRT^46^ with the default parameters (i.e., trilinear interpolation, and unweighted correlation ratio as cost function. The space transformations were applied to all labels (i.e., binary/indexed masks) including manually-derived (i.e., after manually correcting results from a semi-automatic segmentation) labels of WMH. The spatial resolution of the images was 256×256×42 with slice thickness of 0.9375 × 0.9375 × 4 mm. We generated a DEM for each patient by subtracting the manually corrected segmentation of WMH at V1 from the manually corrected segmentation of WMH at V2.

### Data pre-processing for incorporation of probabilistic maps of WMH change

Given the influence of stroke lesion location in WMH change and evolution patterns when the stroke lesions are located at the *centrum semiovale* or the *lentiform nucleus*^20^, we only used probability maps of WMH change based on stroke lesions incident at *centrum semiovale* or *lentiform nucleus*, publicly available from https://datashare.ed.ac.uk/handle/10283/3934.

Probability maps in the standard space were obtained from a clinical study^20^ and then registered to each patient’s native space using niftyreg through TractoR^47^. To identify the location of stroke lesions within a human brain, an age-relevant brain template and its corresponding brain parcellation), also publicly available^48^, were registered to each patient’s native space. If there were no stroke lesions at *centrum semiovale* or *lentiform nucleus* in a patient, then zero matrices were used as probabilistic maps (i.e., there are no specific areas of the brain or feature maps that the neural networks should look for via attention). Both probabilistic maps for *centrum semiovale* or *lentiform nucleus* were concatenated before being used as auxiliary input in the segmentation network (see Fig. 2B for illustration).

### Training scheme

To facilitate comparability between methods and results, we used the same preprocessing pipeline as previous studies^25,26^. To make sure all patients are used in both training and testing and to avoid overfitting, a fourfold cross-validation with 512 epochs was performed, with each fold consisting of 114 MRI for training and 38 for testing. In the training phase, we randomly chose 14 out of 114 MRI training data for validation and used that to select the best model that produced the lowest validation loss (i.e., error difference during training). Values of T2-FLAIR brain MRI were normalised into zero mean and unit variance for each patient. Data augmentations of shifting, scaling, horizontal and vertical flips, and elastic transformations were performed.

### Cost function

We used three lost functions in training to optimize the different networks. These were: (1) segmentation loss (*L*_*seg*_), (2) probabilistic loss using *Kullback–Leibler* Divergence (*D*_*KL*_), and 3) adversarial loss (*L*_*adv*_). We used the segmentation loss to compare the output of the segmentation network (i.e., the predicted DEM segmentation) against the ground truth of the DEM. The probabilistic loss was used to compare the similarity between prior and posterior latent spaces, and the adversarial loss was used to compare the similarity between the ground truth DEM and the predicted DEM.

#### Segmentation loss

For the segmentation loss, we used the weighted focal loss with *γ* (i.e., focal loss’ hyperparameter) set to *γ* = 2 following the recommendation of the original paper^49^. Equation 1 describes the weighted focal loss function for all pixels from an MRI slice where *y*_*i,c*_ ∈ {0*,*1} indicates the class membership for pixel *i* to class *c*, *p*_*i*_ the predicted probability that pixel *i* belongs to class *c*, and *α*_*c*_ is the weight for class *c*. The larger the value of *α*_*c*_, the larger the contribution of class *c* to the loss value. *P* is the random variable for the predicted probability, *Y* is the random variable for the target classes, *α* are the weights for all classes, *N* is the number of pixels in an axial MRI slice (i.e., *N* = 256), and *M* is the number of classes in the DEM (i.e., *N* = 4 if stroke lesions are not automatically segmented and *N* = 5 if otherwise). Based on our preliminary experiments where we performed a grid search, the best weights were *α*_*c*=0_ = 0*.*25 for background, *α*_*c*=1_ = 0*.*75 for shrinking WMH, *α*_*c*=2_ = 0*.*75 for growing WMH, *α*_*c*=3_ = 0*.*5 for stable WMH, and *α*_*c*=4_ = 0*.*75 for stroke lesions.

1 $${\text{FL}}(P,Y,\alpha ) \, = \sum\limits_{i = 1}^{N} {\sum\limits_{c = 0}^{M = 4} {\alpha_{c} y_{i,c} ({1} - p_{i,c} )^{\gamma } log(p_{i,c} )} }$$

Note that the predicted segmentation of the DEM produced by the Probabilistic U-Net is conditioned to either the posterior or the prior latent space. In training, the predicted DEM segmentation is conditioned to the posterior latent space defined by *z*_*post*_ ∼ *N µ*_*post*_*,* *σ*_*post*_ and modelled by the Posterior Net. On the other hand, the predicted DEM segmentation is conditioned by the prior latent space that is formulated as $z_{prior} \sim N \, \mu_{prior} , \, \sigma_{prior}$ and modelled by the Prior Net in testing/inference. Thus, the probabilistic segmentation loss *L*_*seg*_ can be formulated as Eq. 2, where ${Y^\wedge}_{DEM}$ is the predicted DEM segmentation and vol (${Y^\wedge}_{DEM}$*, Y*_*DEM*_) is the newly proposed volume loss to avoid over- and under-segmentation in relation to the future volume of WMH (discussed in the next section), where each loss has the weight of 1 based on preliminary experiments where we conducted grid search.

2 $$L_{seg} = {\text{FL}}(P({Y^\wedge}_{DEM} X_{{V{1},zpost}} ),Y,\alpha ) + {\text{vol}}({Y^\wedge}_{DEM} ,Y_{DEM} )$$

#### Volume loss

To avoid over- and under-segmentation in relation to the future volume of WMH, a volume-loss (that is formulated as Eq. 3) is added to Eq. 2 as regularization term. The term keeps the predicted future volume of WMH (i.e., calculated from the predicted DEM (${Y^\wedge}_{DEM}$)) close to the reference future volume of the WMH (i.e., calculated from the ground truth DEM (*Y*_*DEM*_)) by using mean squared error (MSE). Note that only class *c* = 2 (for growing WMH) and class *c* = 3 (for stable WMH) that matter in the calculation of future volume of WMH at V2. A denominator of 1000 was used to estimate the volume of WMH in *ml* (i.e., as voxel dimensions are in mm^3^). In preliminary experiments, we found that having the same weights for segmentation loss of FL(*P*(${Y^\wedge}_{DEM}$|*X*_*V*1_*,z*_*post*_)*,Y,α*) and volume loss of vol(${Y^\wedge}_{DEM}$*,Y*_*DEM*_) in Eq. 3 produced the best results for predicting both future volume and spatial dynamic changes of WMH.

3 $${\text{vol}}({Y^\wedge}_{DEM} ,Y_{DEM} ) \, = {\text{MSE}}\;\left( {\frac{{\sum\nolimits_{c = 2}^{M = 3} {\hat{y}_{c} } }}{1000},\;\frac{{\sum\nolimits_{c = 2}^{M = 3} {y_{c} } }}{1000}} \right)$$

#### Probabilistic loss

We used *Kullback–Leibler* Divergence score (*D*_*KL*_) in the training process for training the Prior Net and Posterior Net. In this setting, Prior Net and Posterior Net were trained together with the Segmentation Net for predicting the DEM. Let *Q* be the posterior distribution from the Posterior Net and *P* be the prior distribution from the Prior Net. The difference between the posterior distribution *Q* and the prior distribution *P* is described by *D*_*KL*_ in Eq. 4 where *X*_*V*2_ is the T2-FLAIR at V2, *Y*_*DEM*_ is the true DEM, and *X*_*V*1_ is the T2-FLAIR at V1. Based on our preliminary experiments, the dimension for both *z*_*post*_ and *z*_*prior*_ is 4 (smaller than the original paper^27^ which used 6), and the weight for the probabilistic loss is 1.

4 $$DKL(Q||P) \, = {\text{E}}_{zpost} \sim Q,z_{prior} \sim P[{\text{log}}Q(X_{{V{2}}} ,Y_{DEM} ) - {\text{log}}P(X_{{V{1}}} )]$$

#### Adversarial loss

Similar to a previous study^26^, the original adversarial loss proposed by^41^ was slightly modified by adding a segmentation loss (*L*_*seg*_) so that the Segmentation Net was also optimised to produce better segmentation result. Similar to the original paper^41^, the Segmentation Net aims at minimising Eq. 5 while the discriminator network aims at maximising it. In Eq. 5, *G* is the Segmentation Net, *D* is the discriminator network, *y* ∼ (*X*_*V*1_*,X*_*V*2_*,Y*_*DEM*_) is the joint distribution of T2-FLAIR MRI at V1 and V2 and ground truth DEM (i.e., *X*_*V*1_*,X*_*V*2_*,* and *Y*_*DEM*_ respectively), *x* ∼ (*X*_*V*1_*,X*_*V*2_*,*${Y^\wedge}_{DEM}$) is the joint distribution of T2-FLAIR MRI at V1 and V2 and predicted DEM (i.e., *X*_*V*1_*,X*_*V*2_*,* and ${Y^\wedge}_{DEM}$ respectively), E*y* ∼ *Y*_*GAN*_ is the expected value over *Y*_*GAN*_, and E*x* is the expected value over *X*_*GAN*_.

5 $$L_{adv} = {\text{E}}y \sim_{YGAN} [{\text{log}}(D(y))] + {\text{E}}x \sim_{XGAN} [{\text{log}}({1} - D(G(x))) + L_{seg} (G(x))]$$

### Evaluation measurements

In this study, we used the following evaluation measurements to assess the performance of all configurations.

1. ** Volume error** measures how close the predicted WMH volumes are with the real WMH volumes at the follow-up assessment (V2). This is the main performance measurement. Volume error can be calculated by using Eq. 6 where ${\text{vol}}_{true}^{V2}$ is the true volume of WMH at V2, ${\text{vol}}_{predicted}^{V2}$ is the predicted volume of WMH at V2, and ${\text{vol}}_{error}^{V2}$ is the volume error.
  6 $${\text{vol}}_{error}^{V2} = {\text{vol}}_{predicted}^{V2} - {\text{vol}}_{true}^{V2}$$
2. **Accuracy of prediction** assesses how good our proposed models predict WMH evolution for all patients (i.e., growing or shrinking). Accuracy of prediction for growing and shrinking WMH (i.e., subjects with growing and shrinking WMH are correctly predicted to have growing and shrinking WMH respectively) is calculated by the Eqs. 7 and 8 respectively.
  *N*_GRW_ and *N*_SHR_ are the number of subjects in our dataset who have growing and shrinking WMH. Whereas, *P*_GRW_ and *P*_SHR_ are the number of subjects correctly predicted as having growing and shrinking WMH.
  7 $${\text{GRW}} = \frac{{P_{{{\text{GRW}}}} }}{{N_{{{\text{GRW}}}} }}$$
  8 $${\text{SHR}} = \frac{{P_{{{\text{GRW}}}} }}{{N_{{{\text{SHR}}}} }}$$
3. ** Estimated volume interval** (EVI) measures the deviation of the predicted WMH volume at follow-up (V2) from the lowest and highest possible predicted volumes of WMH^26^. The lowest and highest possible predicted volumes of WMH at V2 are estimated by ignoring the prediction channel for growing WMH and shrinking WMH respectively. In other words, the lowest possible volume of WMH (dubbed as Minimum Volume Estimation or ‘MinVE’) is assumed to occur when there are no growing WMH in the patient’s brain. Whereas, the highest possible volume of WMH (dubbed as Maximum Volume Estimation or ‘MaxVE’) is assumed to occur when there are no shrinking WMH in the patient’s brain. There are 3 metrics in this evaluation: “CP” which stands for “Correct Prediction” (calculated by using Eq. 9), “CPinEVI” which stands for “Correct Prediction in Estimated Volume Interval” (calculated by using Eq. 10), and “(CP + WP)inEVI” which stands for “Correct Prediction + Wrong Prediction but still in EVI” (calculated by using Eq. 11). In these equations, $P_{{{\text{GRW}}}}^{in}$ and $P_{{{\text{SHR}}}}^{in}$ are the number of subjects that are correctly predicted as having growing and shrinking WMH and have their estimated volumes of WMH at V2 are located between ‘MinVE’ and ‘MaxVE’. Whereas, *P*^*in*^ is the number of subjects whose estimated volumes of WMH at V2 are located between ‘MinVE’ and ‘MaxVE’.
  9 $${\text{CP}} = \frac{{P_{GRW} + P_{SHR} }}{{N_{GRW} + N_{SHR} }}$$
  10 $${\text{CPinEVI}} = \frac{{P_{GRW}^{in} + P_{SHR}^{in} }}{{N_{GRW} + N_{SHR} }}$$
  11 $${\text{(CP + WP)inEVI}} = \frac{{P_{in} }}{{N_{GRW} + N_{SHR} }}$$
4. **Spearman correlation with Prins clinical scores**: The clinical scoring system for progression of WMH, known as Prins visual scores^39^, gives a + 1 for each WMH cluster that increases or appears *de nuovo* in a subsequent scan compared with a previous scan in the periventricular or deep white matter of each lobe (i.e., frontal, parietal, temporal and occipital),
  -1 if a reduction in volume or disappearance of a WMH cluster is detected, and 0 if no change can be appreciated. For our evaluation, we summed the overall scores in each region to obtain a total Prins score. We calculate the Spearman correlation between the total Prins scores and the spatial volume growth, shrinkage, and overall change that each scheme outputs.
5. **Spatial agreement between predicted and ground truth DEM** is measured by the Dice similarity coefficient (DSC)^50^. Higher values of DSC mean better performance. DSC can be calculated by using Eq. 12, where *TP* is true positive, *FP* is false positive and *FN* is false negative. This is a secondary performance measurement as predicted future WMH volumes at V2 are calculated from segmentation masks.
  12 $$DSC = \frac{2 \times TP}{{FP + 2 \times TP + FN}}$$
6. Uncertainty quantification and correlation analysis to measure correlation between uncertainty values in predicted DEM and DSC values, is calculated as the Cross-Entropy (CE) between the mean sample and all samples as per Eq. 13 where *γ* is the uncertainty map, *s* is a set of predictions from an input, *s*ˆ is the mean sample of set *s*, CE is the cross-entropy function, and E is the expected value function.
  13 $$\gamma ({\text{s}}) \, = {\text{E}}[{\text{CE}}({\text{s}}^{ \wedge } ,{\text{s}})]$$

## Results and discussion

This section shows and discusses the results based on five measurements: predicted future volume of WMH, correlation of future volume of WMH with clinical visual scores, spatial agreement based on DSC, qualitative/visual evaluation, and uncertainty quantification based on CE.

### Results on predicting future volumes of WMH

WMH volume change is an important clinical feature for clinical research and could be an important predictor of recovery after a stroke if available for clinical practice. Hence, we evaluated how well WMH volume at V2 (1 year later) can be estimated using our proposed models. Table 1 shows the prediction accuracy of WMH volumetric progression (i.e., whether WMH volume will grow or shrink at V2 for each patient) calculated using Eqs. 7 and 8 for “GRW” and “SHR”, the estimated volume interval (EVI) calculated using Eqs. 9, 10, and 11 for “CP”, “CPinEVI”, and “(CP+WP)inEVI”, and the volumetric error calculated using Eq. 6 for “Volumetric Error”.

**Table 1:** Volume-based evaluation for all models evaluated. There are 98 patients with growing (GRW) and 54 with shrinking (SHR) volume of WMH. “CP” stands for “Correct Prediction”, “CPinEVI” stands for “Correct Prediction in Estimated Volume Interval”, and “(CP+WP)inEVI” stands for “Correct Prediction + Wrong Prediction but still in EVI”. Symbol ↑ indicates higher values are better, while symbol → 0 indicates that values closer to 0 are better. Each evaluation measurement’s best and second-best values are written in bold and underlined, respectively. PUNet-wSL-vol model is highlighted as it emerged as the best-performing model to estimate the future volume of WMH.

Model’s name	Prediction↑		Estimated volume interval (n = 152) ↑			Volumetric error
	GRW	SHR	CP	CPinEVI	(CP + WP)inEVI	(std) → 0
UNet^43^	79.59%	66.67%	67.11%	48.03%	58.55%	1.267 (8.623)
UNet-vol	80.61%	68.52%	67.11%	46.05%	55.26%	− 0.194 (8.107)
UNet-wSL	72.45%	64.81%	71.71%	38.16%	47.37%	1.038 (9.427)
UNet-wSL-vol	84.69%	59.26%	71.71%	48.68%	59.87%	0.027 (8.662)
PUNet^26^	78.57%	46.30%	67.11%	47.37%	61.18%	− 1.774 (9.798)
PUNet-vol	83.67%	51.85%	71.71%	46.71%	60.53%	− 0.834 (8.657)
PUNet-wSL	75.51%	64.81%	71.71%	48.68%	59.21%	0.227 (10.427)
**PUNet-wSL-vol**	74.49%	74.07%	74.34%	**53.29%**	**62.50%**	− **0.009 (9.751)**
Att-PUNet	70.41%	**79.63%**	73.68%	45.39%	55.26%	3.182 (8.447)
Att-PUNet-vol	81.63%	55.56%	72.37%	43.42%	54.61%	− 0.555 (9.043)
Att-PUNet-wSL	**86.73%**	55.56%	**75.66%**	51.97%	59.87%	− 0.598 (10.901)
Att-PUNet-wSL-vol	81.63%	64.81%	**75.66%**	43.42%	53.95%	0.270 (9.050)

**Fig. 3:**
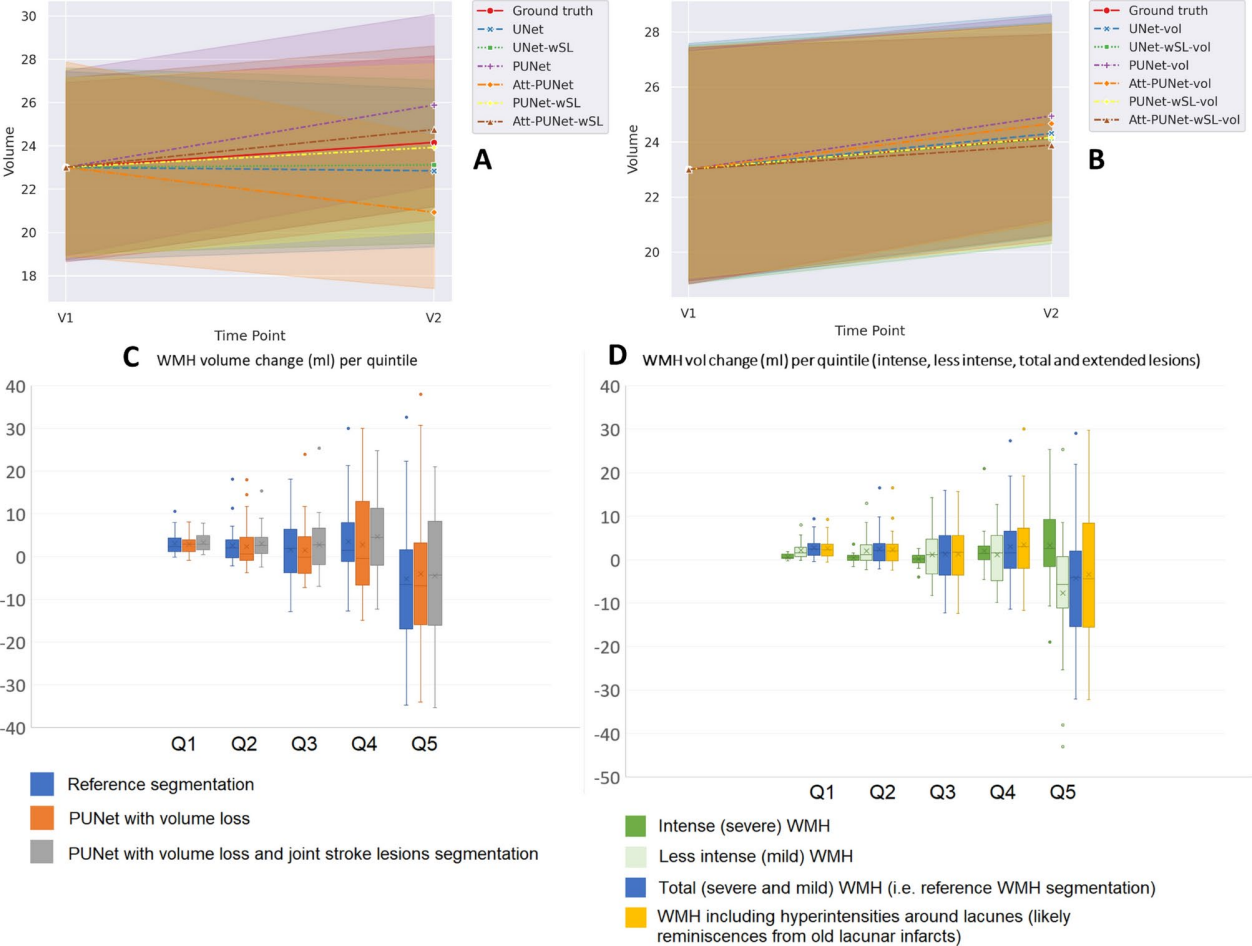
(**A**, **B**) Average progression of WMH volume (ml) from V1 to V2 (1 year) for Ground truth and all tested models/configurations, where (**A**) shows models trained without volume loss and (**B**) shows models trained with volume loss. From panels (**A**) and (**B**), we can see the proposed volume loss’s effectiveness in accurately estimating the future volume of WMH. (**C**, **D**) Volumetric WMH change in ml (vertical axes) for patients grouped by quintiles (horizontal axes) depending on their WMH volume at baseline V1 (i.e., Q1 comprises patients with very small WMH load at V1 (WMH at V1 ≤ 3.01 ml), Q2 includes patients with small WMH load at V1 (3.01 ml < WMH at V1 ≤ 7.56 ml), Q3 includes patients with medium WMH load at V1 (7.56 ml < WMH at V1 ≤ 19.07 ml), Q4 includes patients with large WMH load at V1 (19.07 ml < WMH at V1 ≤ 41.31 ml), and Q5 comprises patients with very large WMH load at V1 (> 41.31 ml)).

As Table 1 shows, PUNet-wSL-vol performed better than the rest of the models, producing either the best or second-best results for almost all evaluation metrics except predicting growing WMH (i.e., GRW). There were more patients with net growing WMH than with net shrinking WMH in the dataset, thus hinting at a possible bias by the other models towards growing WMH. Reduction in WMH volume was mainly observed in patients with high WMH volume (see Fig. 3C).

As Fig. 3B shows, the average progression of WMH volume from V1 to V2 (in *ml*) was well estimated by PUNet-wSL-vol (i.e., the yellow dashed line representing PUNet-wSL-vol is coincident with the red line representing the ground truth). In general, as expected, models trained using volume loss (Eq. 3), shown in Fig. 3B, produced more accurate estimations of WMH volume from V1 to V2 than those that did not use volume loss during training, shown in Fig. 3A. Furthermore, based on the column “Volumetric Error” in Table 1, models jointly segmenting stroke lesions and WMH DEM (i.e., indicated by ‘wSL’ in the “Model’s Name”) improved the estimation of the future volume of WMH at V2.

To further analyse the accuracy of the winner scheme in estimating the WMH volume change, we grouped the patients in quintiles according to their WMH volume at baseline and, then, calculated the WMH change produced by the reference segmentation (i.e., the ground truth) and the PUNet models trained by using volume loss with and without jointly segmenting the DEM and the stroke lesions (Fig. 3C). Hence, the dataset is subdivided for this analysis into five different groups or quintiles (Q) based on the WMH volume at baseline (V1), where Q1 comprises patients with very small WMH load at V1, i.e., WMH at V1 ≤ 3*.*01 ml (i.e., 30 subjects), Q2 includes patients with small WMH load at V1 in the interval 3*.*01 ml < WMH at V1 ≤ 7*.*56 ml (i.e., 31 subjects), Q3 includes patients with medium WMH load at V1, i.e., 7*.*56 ml < WMH at V1 ≤ 19*.*07 ml (i.e., 30 subjects), Q4 includes patients with large WMH load at V1, i.e., 19*.*07 ml < WMH at V1 ≤ 41*.*31 ml (i.e., 31 subjects), and Q5 comprises patients with very large WMH load at V1, i.e., > 41*.*31 ml (i.e., 30 subjects). As can be appreciated from Fig. 3C, the scheme that jointly segmented the stroke lesions and the DEM of WMH change produced mean, median, and distribution of WMH volume change values across the sample more similar to those from the reference segmentation, than the scheme that only segmented the DEM of WMH change for all but the highest quintile.

We also divided the reference WMH segmentations into intense and less intense WMH as per^45^, and considered an ‘extended’ WMH volume that included the WMH surrounding lacunes, thought to be reminiscences of old small subcortical infarcts (see Fig. 3C). It can be observed that the volume output from the scheme that jointly segmented the stroke lesions with the DEM of WMH change resulted strikingly similar to the one produced by this ‘extended’ WMH segmentation (see gray and yellow box plots in Fig. 3B and C, respectively), especially for patients in the highest quintile. Patients in this quintile exhibit a high burden of WMH surrounding lacunes and coalescing with previous strokes. Therefore, it is expected that not only AI schemes but also experts would consider all hyperintensities as part of the white matter disease in the absence of any other sequence or clinical information from this patient group. It can also be seen that the reference WMH change (i.e., blue box plot in the same figure) is mainly determined by the less intense WMH change (i.e., pale green box plot), therefore explaining the difficulty in obtaining accurate growth and shrinking spatial estimates and putting into question the accuracy in the spatial estimates of the ground truth segmentations given the degree of observer-dependent manual input they had.

### Evaluation against clinical visual scores of WMH progression

**Fig. 4:**
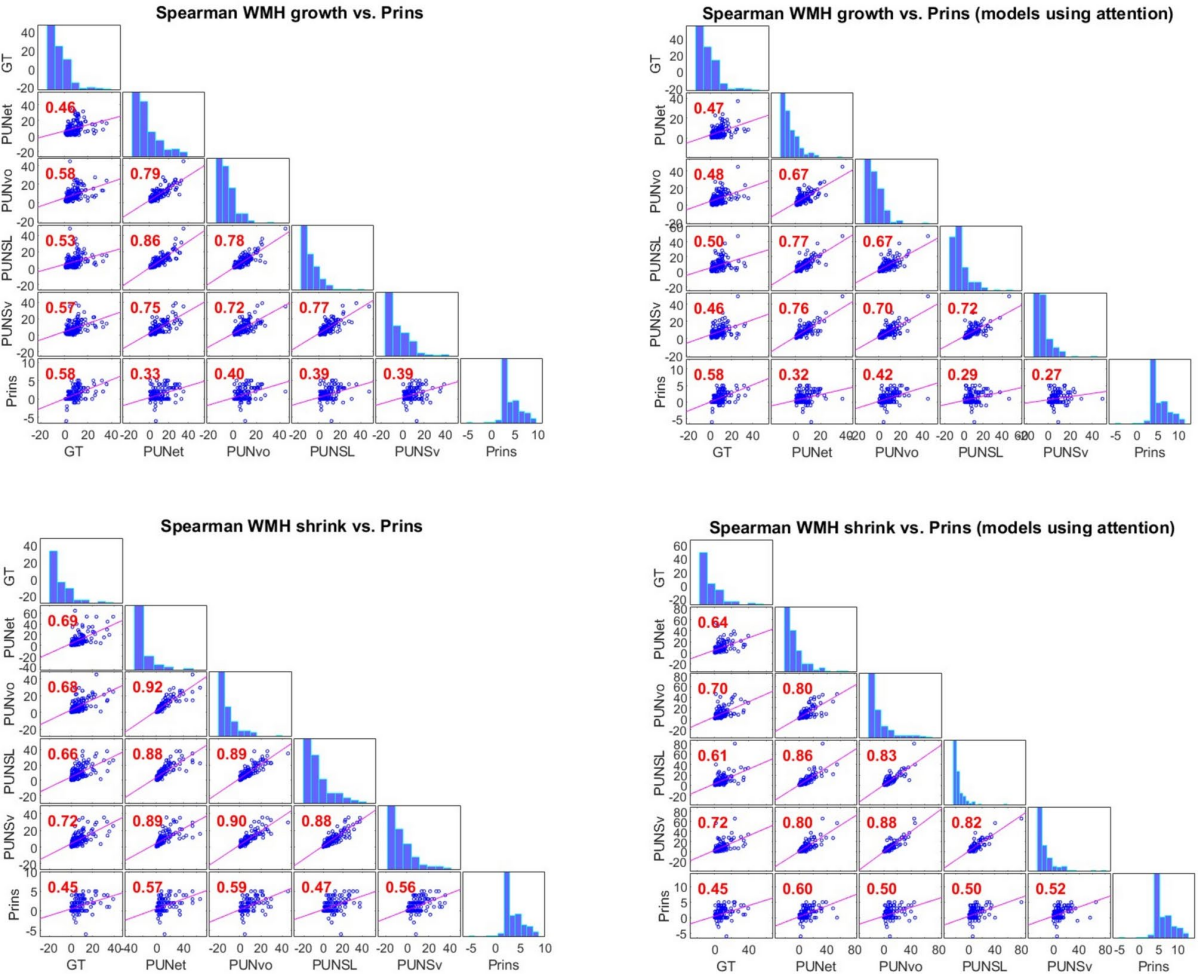
Spearman correlations between the spatial growth in ml (above) and shrinkage in ml (below) from each scheme and the Prins clinical visual overall (summed) scores, presented as blue scatter plots and red Spearman’s ρ values. In these panels, “GT” represents the ground truth, “PUNet” represents the PUNet, “PUNvo” represents the PUNet-vol, “PUNSL” represents the PUNet-wSL, “PUNSv” represents the PUNet-wSL-vol, and “Prins” represents the total (summed) clinical scores of Prins. Panels on the right show results for models that use attention to incorporate stroke lesions information. The bar plots diagonally located in each panel are the histograms representing the distributions of “GT”, “PUNet”, “PUNvo”, “PUNSL”, “PUNSv”, and “Prins”.

Figure 4 shows the results from calculating the non-parametric correlations between Prins clinical visual scores and the spatial volume growth and shrinkage from each Probabilistic U-Net scheme. The spatial growth from all models correlated with Prins scores, with the output from PUNet-vol showing the highest correlation following the ground truth (Spearman’s *ρ* = 0.40 and 0.58, respectively). This correlation slightly improved (i.e., to Spearman’s *ρ* = 0.42) when attention was incorporated in the scheme. Prins showed net shrinkage for only six patients, as shrinkage in individual clusters were nullified by growth in others. The ground truth showed the worst correlation with Prins in terms of shrinkage (Spearman’s *ρ* = 0.45), followed by PUNet-wSL (Spearman’s *ρ* = 0.47 without attention and 0.50 with it). The highest correlation values in shrinkage were achieved with PUNet-vol without attention (Spearman’s *ρ* = 0.59), and PUNet with attention (Spearman’s *ρ* = 0.60). In general, in models without attention, WMH shrinkage and growth correlated better with Prins than when attention was used. In line with a previous study^51^, the spatial net change did not correlate with Prins, neither improving when attention was used.

### Spatial agreement evaluation

We evaluated spatial agreement to see whether the predicted future volumes of WMH closer to the reference future WMH volumes are followed by higher spatial agreements between predicted DEM and ground truth DEM or not. Table 2 shows performances of all tested configurations using DSC (Eq. 12). The best and second-best measurement values for each DEM label are written in bold and underlined, respectively. Note that the ‘Changing’ refers to shrinking and growing WMH combined together as one label, ‘Average’ is calculated by averaging DSC values of ‘Shrinking’, ‘Growing’, and ‘Stable’, and ‘Stroke Lesions’ is only available when joint segmentation of WMH DEM and stroke lesions are performed.

From Table 2, we can see that joint segmentation of DEM and stroke lesions with volume loss (PUNet-wSL-vol) produced the best segmentation results based on DSC for ‘Shrinking’ (0.2290). Furthermore, we can see that joint segmentation of DEM and stroke lesions by PUNet-wSL (i.e., without volume loss) and PUNet-wSL-vol (i.e., with volume loss) produced either the best or second-best DSC values for ‘Changing’ WMH (i.e., the combination of ‘Shrinking’ and ‘Growing’ WMH) than the original U-Net, which performed better on segmenting ‘Stable’ WMH. This leads to PUNet-wSL-vol’s better performance in estimating the future volume of WMH, as shown in Table 1. On the other hand, models with auxiliary input of probabilistic maps of WMH change (i.e., Att-PUNet, Att-PUNet-vol, Att-PUNet-wSL, and Att-PUNet-wSL-vol) failed to improve the performance of the DEM segmentation while improving the performance of ‘Stroke Lesions’ segmentation. Furthermore, models trained using volume loss (i.e., UNet-wSL-vol, PUNet-vol, Att-PUNet-vol, PUNet-wSL-vol, and Att-PUNet-wSL-vol) produced better DSC values on ‘Average’, which indicates that the volume loss impacted positively in the task of estimating the DEM of WMH.

**Table 2:** Dice similarity coefficient (DSC) for all model configurations. Symbol ↑ indicates that higher values are better. The best values from each measurement are written in bold and and second-best values are underlined. As can be appreciated, in this evaluation, the PUNet-wSL models performed better in segmenting changing WMH (i.e., the combination of ‘Shrinking’ and ‘Growing’ WMH) than the original U-Net, which performed better on segmenting ‘Stable’ WMH. PUNet-wSL-vol had an overall better performance in estimating the future volume of WMH, as per Table 1.

Model’s name	Shrinking	Dice similarity coefficient (DSC) ↑				Stroke lesions
		Growing	Stable	Average	Changing	
UNet^43^	0.2228	0.2077	**0.6609**	**0.3638**	0.3644	–
UNet-vol	0.2239	0.2155	0.6485	0.3626	0.3649	–
UNet-wSL	0.2093	0.2026	0.6420	0.3513	0.3499	0.3588
UNet-wSL-vol	0.2125	0.2189	0.6452	0.3589	0.3579	0.3422
PUNet^26^	0.2132	0.2137	0.6385	0.3551	0.3633	–
PUNet-vol	0.2107	**0.2232**	0.6439	0.3593	0.3642	–
PUNet-wSL	0.2217	0.2130	0.6437	0.3595	**0.3719**	0.4499
PUNet-wSL-vol	**0.2290**	0.2112	0.6392	0.3598	0.3681	0.4281
Att-PUNet	0.2211	0.1796	0.6302	0.3437	0.3510	–
Att-PUNet-vol	0.2078	0.1981	0.6315	0.3458	0.3471	–
Att-PUNet-wSL	0.1968	0.2045	0.6240	0.3417	0.3543	0.5338
Att-PUNet-wSL-vol	0.1960	0.2077	0.6322	0.3453	0.3536	**0.5430**

DSC is influenced by TP, FP, and FN counts between ground truth mask and predicted segmentation, but TP, FP, and FN counts are highly imbalance in the segmentation of brain lesions. To provide a better illustration of the relationship between

**Fig. 5:**
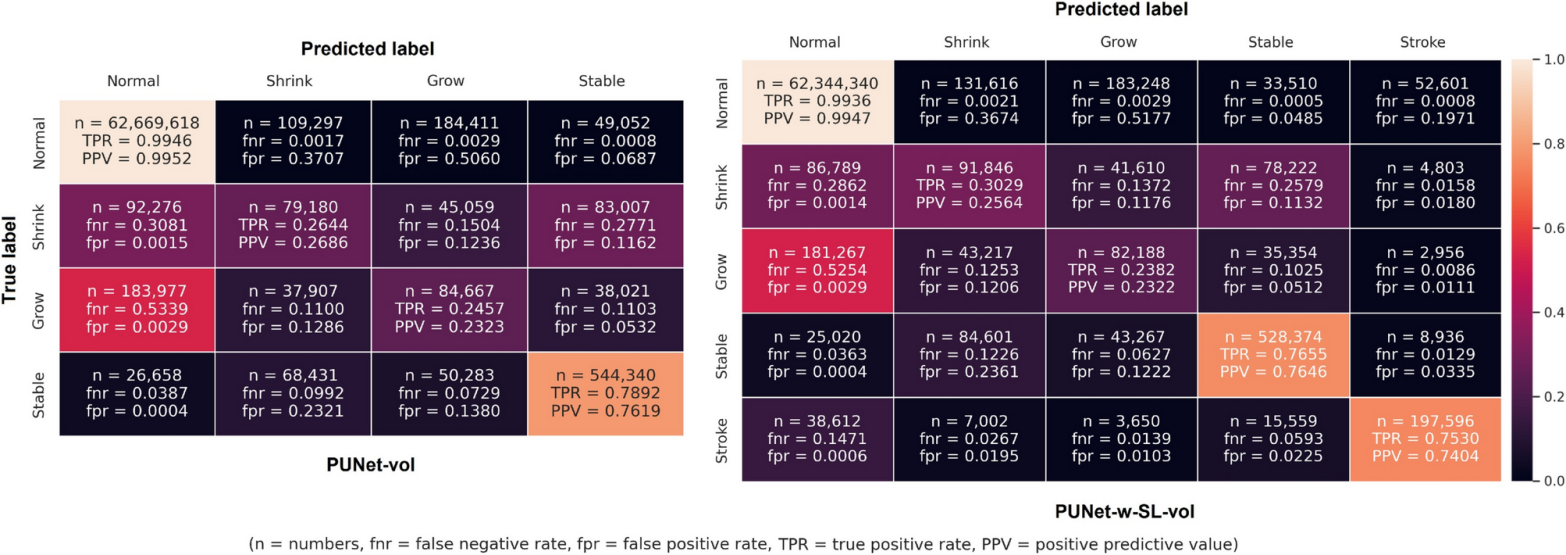
Confusion matrices for all labels produced by PUNet-vol and PUNet-wSL-vol configurations from all subjects. Abbreviation n stands for number of segmented voxels which can be used to calculate false negative rate (fnr), false positive rate(fpr), true positive rate (TPR), and positive predictive value (PPV). Note that TPR and fnr are calculated horizontally for each row (true label of DEM). On the other hand, PPV and fpr are calculated vertically for each column (predicted label of DEM).

DSC and corresponding TP, FP, and FN counts, we present the confusion matrices and a table compiling these values from the ‘Shrinking’ WMH and ‘Growing’ WMH labels obtained from PUNet-vol and PUNet-wSL-vol configurations (Fig. 5 and Table 3 respectively). Fig. 5 contains the number of segmented voxels corresponding to each label (*n*) from all patients in the testing set, false negative rate (*fnr*), false positive rate (*fpr*), true positive rate (TPR), and positive predictive value (PPV). Table 3 compiles values of DSC, PRE, REC, FN, and FP for the ‘Shrinking’ WMH and ‘Growing’ WMH labels from both PUNet-vol and PUNet-wSL-vol configurations. From both, Fig. 5 and Table 3, we can see that PUNet-vol produced higher PRE value for ‘Shrinking’ WMH with lower FP counts than PUNet-wSL-vol. But PUNet-vol produced lower PRE value for ‘Growing’ WMH as it produced higher FP counts than PUNet-wSL-vol in this label/category.

**Table 3:** Comparison of DSC, PRE, and REC values to FN and FP counts for PUNet-vol and PUNet-wSL-vol configurations. Symbols ↑ and ↓ indicate that higher and lower values are better respectively.

	Shrinking WMH					Growing WMH				
	DSC ↑	PRE ↑	REC ↑	FN ↓	FP ↓	DSC ↑	PRE ↑	REC ↑	FN ↓	FP ↓
PUNet-vol	0.2107	0.2527	0.2408	220,342	215,635	0.2232	0.2391	0.2569	259,905	279,753
PUNet-wSL-vol	0.2290	0.2295	0.3066	211,424	266,436	0.2112	0.2479	0.2346	262,794	271,775

Confusion matrices in Fig. 5, show a high level of uncertainty between ‘Growing’ WMH and ‘Normal’ brain tissues as more than 50% of the ‘Growing’ WMH identified in the ground truth DEM were wrongly predicted as ‘Normal’ tissues (i.e., under-segmentation of ‘Growing’ WMH which leads to higher *fnr* in the confusion matrix) by PUNet-vol and PUNet-wSL-vol configurations with *fnr* = 0*.*5339 and *fnr* = 0*.*5254 respectively. In extended experiments, all proposed configurations were observed producing the same level of under-segmentation for ‘Growing’ WMH. In general, areas of ‘Growing’ WMH are difficult to differentiate from ‘Normal’ brain tissues due to the high level of uncertainty between these two classes. Overall, for the model that jointly segmented the stroke lesions and the WMH, mean DSC values were slightly better in this sample.

Although the combined segmentation of WMH and stroke lesions is not the main focus of this study, it must be noted that the state-of-the-art joint segmentation method for WMH and stroke lesions (i.e., sub-acute and chronic as per in the present dataset)^52^, which used a UResNet configuration, reported a mean (SD) Dice equal to 0.4 (0.252) for stroke lesions segmentation, lower than any of our joint-segmentation schemes (see Table 2).

### Qualitative/visual evaluation of spatial agreement between ground truth and predicted DEMs

**Fig. 6:**
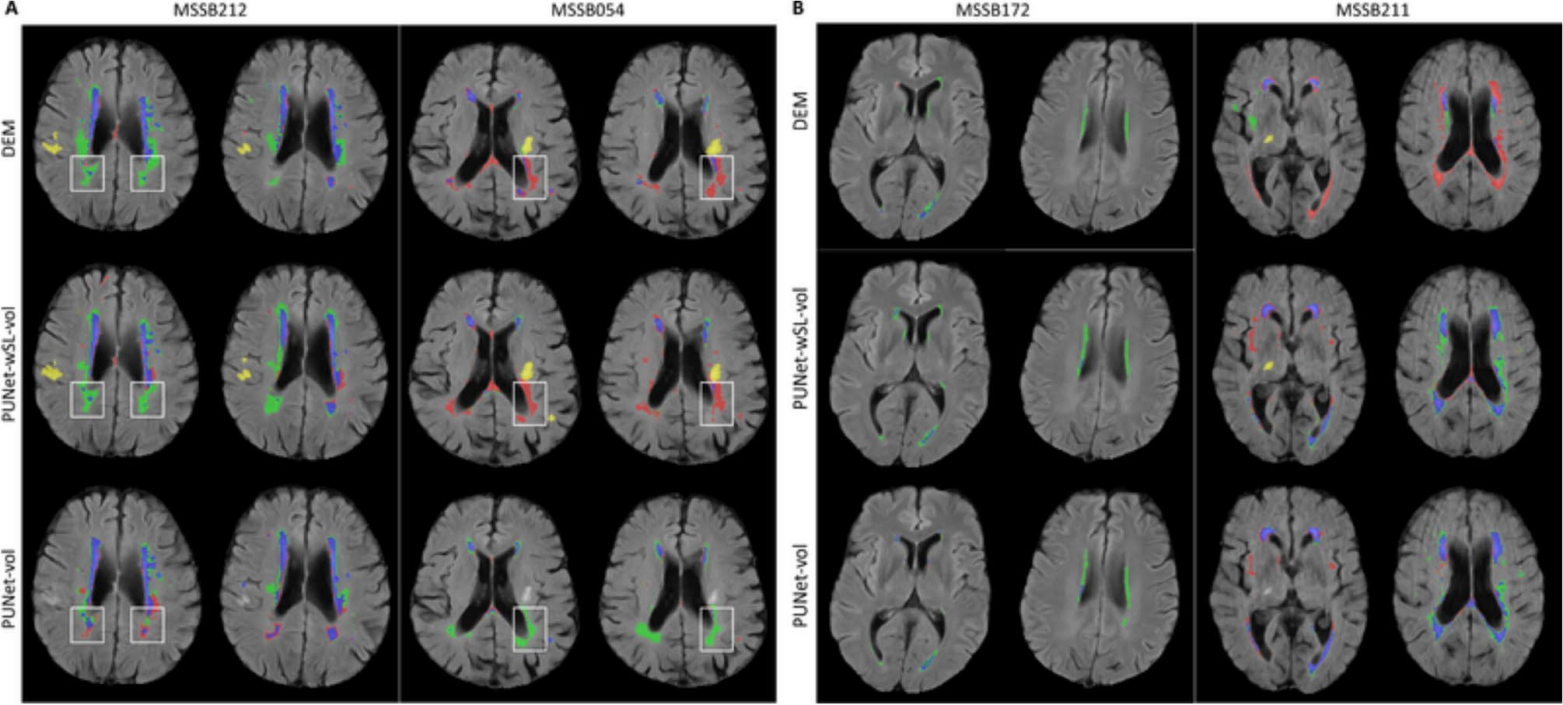
(**A**) Examples of predicted DEM produced by PUNet-wSL-vol and PUNet-vol and their corresponding DEM ground truth from subjects with high DSC values on average. (**B**) Examples of predicted DEM produced by PUNet-wSL-vol and PUNet-vol and their corresponding DEM ground truth from subjects with low DSC values on average. (**A**, **B**) Red represents shrinking WMH, green represents growing WMH, blue represents stable WMH, and yellow represents stroke lesions. Obvious improvements are highlighted with white rectangles.

Figure 6A and B show examples of the predicted DEM segmentation from PUNet-wSL-vol and PUNet-vol and their corresponding DEM ground truth forpatients with high and low DSC values on ‘Average’ respectively. PUNet-wSL-vol and PUNet-vol were chosen for qualitative/visual evaluation as they produced the best and second best DSC values on ‘Average’ (See Table 2). Figure 6A shows that PUNet-wSL-vol, which jointly segments WMH DEM and stroke lesions, produced better segmentation results than PUNet-vol, which exhibits a high level of uncertainty in predicting shrinking and growing WMH. Confusion matrices in Fig. 5 show that PUNet-wSL-vol lowered this uncertainty by producing lower rates of *fnr* (and their corresponding FN counts (*n*)) for shrinking and growing WMH) in most cases. Figure 6B illustrates cases where low DSC values of predicted WMH DEM were caused mostly by two reasons: low WMH volume at V1 (patient and MSSB172) and brain MRI artefacts (patient MSSB211). Based on our observations, these two problems were relevant throughout the sample in our evaluations.

### Uncertainty quantification

As all configurations evaluated are based on the Probabilistic U-Net, uncertainty for each label in the DEM was quantified by predicting DEM for each subject multiple times. In this study, 30 different DEM predictions were generated from 30 samples of *z*_*prior*_ from Prior Net for each input data/patient. From these 30 DEM predictions per patient data, uncertainty was calculated as the Cross-Entropy (CE) between probability values from all DEM predictions and its average as written in Eq. 13.

**Fig. 7:**
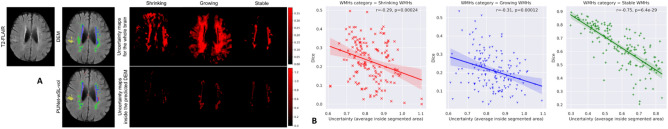
(**A**) Uncertainty maps produced by PUNet-wSL-vol from subject MSSB212. (**B**) Correlation between the average of uncertainty values inside the predicted DEM and DSC values of the predicted DEM produced by PUNet-wSL-vol.

Figure 7 shows the uncertainty maps for all DEM labels produced by the model that generated the best DSC ‘Average’ value, PUNet-wSL-vol, for the whole brain and inside the predicted DEM for a patient. From the uncertainty maps for the whole brain, we can see that the uncertainties for shrinking and growing WMH encompass larger brain areas than for stable WMH. This finding supports results from evaluating the spatial agreement between ground truth and the models’ outputs, indicating lower accuracy in the predictions of ‘Changing’ WMH (i.e., ‘Shrinking’ and ‘Growing’ WMH) than the predictions of ‘Stable’ WMH. The example shown in Fig. 7A has incorrect areas showing uncertainty in the ‘Shrinking’ label (e.g., in the frontal cortex and the septum), owed mainly to hyperintense flow artefacts.

Interestingly, in the uncertainty maps, the uncertainty values inside DEM labels of shrinking and growing WMH are higher than those inside stable WMH, a consistent finding from this evaluation. This is in-line with a previous analysis^51^ that showed WMH progression and disappearance being associated with the areas of ill-defined subtle or “less intense” WMH, largely identified as indicative of pre- (and post-) lesional changes. As expected, Fig. 7B shows that the uncertainty values inside the predicted DEMs and the DSC values produced by PUNet-wSL-vol are negatively correlated for each DEM label (i.e., ‘Shrinking’, ‘Growing’, and ‘Stable’ WMH). However, only for the ‘Stable’ WMH (r = 0.75) can higher DSC values of DEM labels be predicted by having lower uncertainty values inside the predicted DEM and vice versa. Plots of the correspondence in shrinking and growing labels show a wide spread in DSC values especially among those with uncertainty values between 0.7 and 0.98, that would make any inference of the predictive power of one magnitude over the other inaccurate.

## Conclusion

This study proposed deep learning models that incorporate stroke lesions information based on the Probabilistic U-Net architecture^27^ with adversarial training^26^ trained by using additional volume loss for improving the quality of predicted future volume of WMH and disease evolution map (DEM) of WMH. Probabilistic U-Net was chosen as the baseline method because a preliminary study showed that it performed better than the U-Net^26^.

We proposed three different approaches for incorporating stroke lesions information into Probabilistic U-Net models. These are (1) joint segmentation of DEM and stroke lesions, (2) use of probabilistic maps of WMH change in relation to stroke lesions’ locations, and (3) combination of (1) and (2). We proposed to incorporate stroke lesions information into deep learning models to predict WMH evolution because stroke is commonly associated with the evolution of WMH^3^. Based on the results from the various experiments, joint segmentation of DEM and stroke lesions (approach (1)) was the most effective approach to improve the quality of predicted DEM of WMH in all evaluations while also being simpler and more straightforward than the other approaches evaluated in this study. The introduction of a volume loss as an additional loss to the scheme substantially improved the quality of predicting the DEM of WMH in terms of the future volume of WMH, correlation with clinical scores of WMH progression, and spatial agreement in DSC.

This study shows that (1) incorporating factors that have been commonly associated with WMH progression (i.e., stroke lesions information) is crucial to produce better prediction of DEM for WMH from brain MRI; (2) the best method for incorporating associated factors that can be extracted from the same data/image modality involves performing multi-task learning; and 3) in patients with vascular pathology, a multi-class segmentation of brain features resulting from symptomatic (i.e. stroke) and asymptomatic (i.e., WMH) vascular events generates better results consistent with clinical research. In this study, as stroke lesions appear on the same T2-FLAIR MRI sequence as WMH, we performed joint segmentation of DEM for WMH and stroke lesions. However, previous clinical studies have shown that there are other non-image risk factors and brain features that have been commonly associated with the progression and evolution of WMH, like age^8^, ventricular enlargement^53,54^, and brain atrophy^55^. Thus, more (image and non-image) factors could be incorporated in future studies to further improve the quality of predicted DEM of WMH, although the best way to incorporate non-image factors to the prediction model remains to be found.

This study also has limitations to overcome in future works. The dataset was small in size, impeding a quantitative in-depth analysis of the models’ performance in different patient subgroups, e.g., patients stratified by age and sex, patients grouped by stroke subtype, etc. Thus, subgroup analyses were carried out visually and volumetrically, not spatially. By using only data from patients presenting to a clinic with a mild-to-moderate stroke, the generalisability of the proposed approach can be questioned. Therefore, further evaluation in a wider and more heterogeneous sample will be needed. The use of DSC in the evaluation needed the binarisation of the probabilistic outputs from the models. Limitations in using DSC have been recently acknowledged^56^. However, it must be noted that ground truth segmentations are also binary and observer-dependent. By using different quality control metrics in a comprehensive analysis, we have overcome the limitations posed by analyzing the spatial agreement using DSC. A probabilistic metric allowing spatial analyses of segmentation results is needed. Also, we used probabilistic maps of WMH change for strokes in the lentiform nucleus and centrum semiovale based on findings from a clinical study. However, the same clinical study specified that it was not possible to ascertain WMH evolution and distribution for patients with strokes in other regions like the thalami and midbrain or brain stems due to the limited sample of patients with infarcts in those regions. Incorporating findings for more powered studies would be necessary to conclude the usefulness of incorporating attention maps into AI schemes. Finally, various schemes for estimating uncertainty in segmentation/classification tasks have recently emerged^57,58^, which would be worth exploring in the future for estimating WMH evolution.

## Additional information

Accession codes: The training codes are publicly available on https://github.com/febrianrachmadi/probunet-gan-vie. Whereas inference models based on the Docker engine are publicly available on https://github.com/febrianrachmadi/ wmh-prediction.
